# Efficacy of auditory gamma stimulation for cognitive decline: a systematic review of individual and group differences across cognitively impaired and healthy populations

**DOI:** 10.1038/s41514-025-00305-1

**Published:** 2025-12-05

**Authors:** Eve Bolland, Aoibhe De Burca, Sheng Hsuan Wang, Alexander Khalil, Gráinne McLoughlin

**Affiliations:** 1 Social, Genetic & Developmental Psychiatry Centre, Institute of Psychiatry, Psychology & Neuroscience, King’s College London, London, UK; 2 Department of Music, School of Film, Music, and Theatre, University College Cork, Cork, Ireland

**Keywords:** Neurology, Neuroscience

## Abstract

Auditory gamma stimulation is a promising non-invasive neuromodulation technique for cognitive decline, with preclinical studies demonstrating therapeutic effects in Alzheimer’s disease models. However, translating these findings into human trials has produced variable outcomes, suggesting a need to examine factors influencing efficacy. In a systematic review of 62 studies on healthy and cognitively impaired populations, we identified 16 characteristics that may affect the response to stimulation. Outcomes reported included improved cognition, slower progression of brain atrophy, and changes in functional connectivity. Optimal stimulation frequency varied across individuals, indicating that personalised approaches may be valuable. Importantly, animal-model findings regarding amyloid clearance and reduced neuroinflammation were not consistently replicated in human studies, nor did neurophysiological responses reliably predict cognitive or biological effects. Significant methodological diversity was evident, with 32 neurophysiological measures employed, highlighting a need for standardisation. Future research should prioritise consensus on outcome measurement and explore individualised intervention strategies to better assess therapeutic potential.

## Introduction

Dementia currently affects an estimated 57 million individuals worldwide, with projections indicating a rise to 75 million by 2030 and 152 million by 2050^1^. While pharmacological treatments for dementia are on the rise, they have yet to provide effective solutions for all individuals, prompting increased interest in alternative approaches such as non-invasive brain stimulation. Among the various causes of dementia, Alzheimer’s disease (AD) accounts for 60–80% of cases and is characterised by distinct alterations in neural gamma oscillatory dynamics^2,3^ including impaired cross-frequency coupling, and disruptions in network-level gamma coherence^4^.

Gamma oscillations (30–80 Hz) arise primarily from interactions between fast-spiking parvalbumin-expressing gamma-aminobutyric acid (GABA)-ergic interneurons and excitatory pyramidal neurons and support both local processing and interareal coherence^5–8^. Measured by electroencephalography (EEG)^9^ or magnetoencephalography (MEG)^10^, these oscillations contribute to essential cognitive functions including attention, visual processing, working memory, reasoning, and executive function^11–14^, while disruptions in gamma activity have been implicated in various neuropsychiatric disorders^15–19^.

Brain oscillations can be modulated through repetitive stimulation, a process known as entrainment, which aligns oscillations to an external rhythm^20^. Given the link between aberrant gamma oscillations and cognitive dysfunction^3,21^, entrainment is proposed to improve cognition by restoring normal gamma activity^22^ and enhancing neural processing efficiency^23^. Various non-invasive methods have been employed to induce gamma entrainment, including transcranial electrical or magnetic stimulation^24,25^ and sensory stimulation, such as flickering light (visual)^26^ or periodic clicks (auditory)^27^, with sensory methods offering superior comfort and ease of use^28,29^. Research has found that gamma entrainment via these means has been associated with enhanced performance in motor processes^30^, perception^31^, attention^32^, and memory^33,34^ in healthy individuals. Importantly, gamma oscillations and the cognitive functions they support are disrupted in AD, suggesting that gamma entrainment may represent a potential therapeutic to restore aberrant oscillatory activity and, thus, the associated cognitive processes^16^.

Consequently, the potential of sensory gamma entrainment as a therapeutic for cognitive decline in dementia, specifically in AD, is rapidly gaining traction. In a seminal study, Iaccarino et al.^35^ demonstrated that visual entrainment of 40-Hz oscillations in the hippocampus of AD mouse models significantly reduced amyloid-beta (Aβ) levels, a hallmark of AD pathology^36,37^, and activated microglia, suggesting enhanced amyloid clearance through increased endocytosis and reduced amyloidogenesis^38,39^. Subsequent studies using 40-Hz sound and light stimulation have largely corroborated these findings, showing not only reduced Aβ deposition extending beyond the sensory cortices^40,41^ but also broader neuroprotective effects, including improved synaptic function, enhanced neuronal integrity, reduced inflammation and reversal of deficits in long-term potentiation, which is critical for learning and memory^42,43^.

The promising results from animal studies have catalysed a surge of clinical trials exploring the therapeutic potential of gamma entrainment in AD. While preclinical models demonstrate reductions in amyloid pathology and neuroinflammation^35,42,43^, translating these findings to humans has proven to be more complex. A recent review examines the differences between preclinical and clinical findings, noting that while animal models show robust effects on pathology and cognition, clinical trials demonstrate mixed outcomes, with only modest effects observed to date in some human studies^44^. The precise mechanisms by which sensory stimulation interacts with neural oscillations and AD pathology require further investigation and likely vary across brain regions and disease stages.

One explanation for the inconsistent replication of neuroprotective effects of sensory gamma stimulation in humans may stem from individual and group differences in responsiveness to neuromodulation^45^. Factors such as neurotransmitter balance, brain state, age, and sex influence the response to electrical neuromodulation^46,47^, with neuroanatomical and neurophysiological variability significantly predicting its efficacy^48^. Indeed, age has been associated with changes in evoked gamma and altered neural dynamics, possibly reflecting structural brain changes and shifts in neurotransmitter function^49–51^. Importantly, both electrical and magnetic neuromodulation have been shown to elicit stronger neural effects in younger individuals, with comparatively attenuated responses observed in older populations^52,53^. Similarly, sex differences in brain structure and functions, including greater cortical thickness^54^, hormonal fluctuations^55^, and variations in neurotransmitter levels^56^, may contribute to stronger and more synchronised gamma oscillations in females compared to males^57,58^.

Importantly, disease-related factors may influence gamma oscillations and the neuromodulatory response. AD, in particular, has been associated with reduced gamma power and synchronisation at rest^59–61^. While neuromodulatory techniques have demonstrated improvements in cognitive performance in mild cognitive impairment (MCI)^62,63^, one review highlights that, across diverse gamma neuromodulation approaches, clinical research in AD remains limited, with small-sample trials reporting variable but some positive cognitive outcomes^64^. Variable outcomes of neuromodulation may relate to atrophic changes which progressively increase with disease severity^65^. Damage to white matter tracts in late-stage neurodegeneration^66^ impairs signal propagation, even with successful entrainment in localised regions^67^. It may therefore be important to consider individual differences in the context of disease stage when applying neuromodulatory interventions to patients with AD.

Consequently, personalised approaches have been applied in some neuromodulation techniques, such as repetitive transcranial magnetic stimulation (rTMS), where parameters are adjusted to maximise efficacy for each individual^68^. Yet, the influence of individual differences on treatment outcomes with sensory gamma entrainment remains largely unexplored.

This systematic review examines whether specific groups and/or individuals exhibit distinct responses to auditory gamma stimulation to consider how these variations may influence downstream cognitive and biological effects, with a view towards preventing cognitive decline. There is a notable gap in the literature bridging preclinical and clinical findings in sensory stimulation, with limited exploration of individual variability in human studies. Understanding both individual and group-level factors is essential, especially as research on sensory gamma entrainment for neurodegeneration rapidly expands. As auditory stimulation appears the most effective sensory modality for entrainment when delivered alone, we focus our search on auditory stimulation, including studies that incorporate simultaneous visual stimulation (audiovisual) which has been shown to enhance the entrainment response^69,70^.

To address this knowledge gap, our review has three objectives. First, to examine how individual characteristics influence the effectiveness of auditory (and audiovisual) gamma stimulation for entrainment in the healthy population and the associated effects on cognition and behaviour. Second, evaluate how the efficacy and outcomes of auditory gamma stimulation differs between neurological conditions associated with cognitive decline and healthy populations. Finally, explore how individual characteristics interact with clinical conditions in response to auditory gamma stimulation to influence therapeutic outcomes.

## Results

**Fig. 1:**
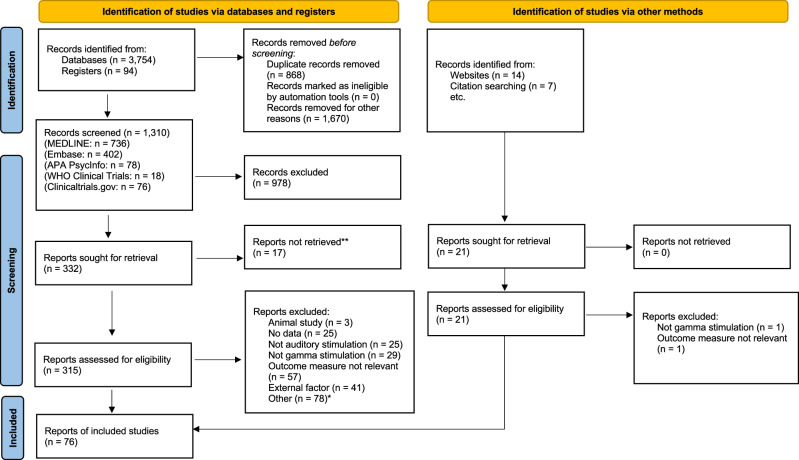
Study Identification PRISMA Flow Diagram. *Other reasons for exclusion included insufficient information reported for data extraction, duplication of studies under different titles (e.g., preprint and published version), and clinical conditions outside the scope of the current review, such as psychiatric disorders or loss of consciousness. **Papers were also excluded where the full text could not be retrieved despite searching across databases and other online sources.

The initial search retrieved 3,754 records from the databases included and 94 records from the clinical trial registers (Fig. 1). Following limits and the removal of duplicate records, the database search was reduced to 1209 reports. This search was reconducted one month before data extraction for inclusion in the final sample to capture any newly added records and resulted in an additional seven records, amounting to 1310 records for screening. At this stage, the manual citation searching and AI search were conducted, yielding an additional 21 records for screening. Following title screening, abstracts and full texts of 336 records were assessed for eligibility. Of these, 76 met the inclusion criteria: 14 were clinical trials that were either ongoing or available only as abstracts (included in Supplementary Data 6) to highlight current studies investigating auditory gamma stimulation as an intervention for neurological conditions associated with cognitive decline. The remaining 62 studies were included in the main synthesis.

Reasons for exclusion included non-human samples, records without data included (e.g. review papers), designs using a different sensory modality or frequency band for stimulation, studies with outcome measures not relevant to the topics at hand, studies investigating the influence of an external factor (e.g. stimulus intensity) on the entrainment response, duplication of studies under different titles (e.g. preprint and published version), and clinical conditions outside the scope of the current review (e.g. psychiatric disorders). Sixteen records were additionally excluded as they consisted of a conference abstract only, meaning quality assessment could not be conducted, and many overlapped with later published articles included in the review. Across the 62 studies included in the main synthesis, a total of 2179 participants were examined. Sample sizes ranged from four to 181 participants. Study designs comprised 11 observational, 43 experimental, two longitudinal, and six clinical trials. Participant ages ranged from three months to 75 years.

Four key themes emerged from the studies selected for data extraction: Theme 1) individual differences affecting the entrainment response in healthy populations, and sub-theme 1a) neurodevelopmental conditions affecting entrainment (Table 1 (Supplementary Data 1), Table 1a (Supplementary Data 2) respectively); Theme 2) clinical conditions affecting the entrainment response (Table 2, Supplementary Data 3); Theme 3) cognitive and biological effects of entrainment in healthy and clinical populations (Table 3, Supplementary Data 4); Theme 4) optimisation of stimulation frequency in healthy and clinical populations (Table 4, Supplementary Data 5). Outcome measures in Themes 1, 1a, 2, and 4 comprise neurophysiological measures of entrainment, typically EEG measures, with a note included where MEG was instead (Tables 1, 1a, 2, and 4). While studies investigating gamma stimulation in individuals with neurodevelopmental conditions do not strictly fall under our predefined categories of healthy individuals or those with cognitive decline, there was no strong rationale to exclude them. In line with the growing recognition that ADHD and ASD represent neurodevelopmental differences rather than inherently “unhealthy” conditions, we treated these populations as reflecting trait-level variability relevant to entrainment. They have been included as a sub-theme of Theme 1. Outcome measures for Theme 3 comprise measures of cognitive function including task performance or clinical assessments, and measures of biological change, including both neurobiological measures (e.g., brain atrophy, functional connectivity) and physiological responses (e.g., sleep quality, adverse events) (Table 3). Stimulation protocol details are provided in the tables.

**Table 1:** Overview of individual differences affecting entrainment in healthy populations

Author (Year)	Country	N	Mean Age	Trait(s)	Stimulation (Hz)	Outcome Measures	Key Findings
Lorenzini et al.^91^	France	45	3 months for the 3-month-old infants, 9 months 22 days for the 10-month-old infants, 23.2 years for the young adults	Age	8, 40	AMFR, SNR	Adults showed consistent AMFR at 40 Hz, infants showed almost no response
Arutiunian et al.^78^	Russia	30	9.1	Age	40	ITPC, ERF amplitude (MEG)	ITCP increased with age. ERF amplitude decreased with age. Stronger ITPC was associated with lower ERF amplitude
Ross & Fujioka ^93^	Canada	24	Younger adults: 23.1 Older adults: 67.8	Age	40	Amplitude, temporal dynamics (MEG)	In quiet conditions, younger participants exhibited a larger 40-Hz response, while under noise masking, amplitudes were similar. Older adults showed delayed recovery in 40-Hz oscillations following stimulus gaps
Cho et al.^82^	USA	181	14.5	Age	20, 30, 40	Power, PLF, CFC	Power, PLF, and CFC increased with age from 8 to 16 y and decreased from 20 to 22 y for 40 Hz. All measures remained flat or decreased for 20 and 30 Hz from childhood to early adulthood
Griškova-Bulanova et al.^71^	Lithuania	46	Not reported. Range: 20-58	Age	40	PLI, amplitude, total intensity	PLI and evoked amplitude diminished linearly with age, no effect of age on total intensity
Johnson et al.^83^	Canada	12	38 (younger), 69.6 (older)	Age	40	Phase, amplitude	No differences found between age groups
Rojas et al.^86^	USA	69	25.62	Age, sex	Not specified	Power, amplitude (MEG)	Peak response at 40 Hz, no age-related shift in peak frequency (all around 39.8 Hz). Power increased with age, stabilising in early adulthood, with no sex differences. Variability in amplitude increased with age
Roth et al.^92^	USA	8	Range: 21-40	Attention	40	SNR	40-Hz response increased when difficulty of task decreased, and in passive condition when concentrating on auditory stimuli
Griškova-Bulanova et al.^85^	Lithuania	27	23.2	Attention	40	GFS	Global synchronisation weakened with distraction from stimulation
Alegre et al.^80^	Spain	12	27.6	Attention	1-120	ITC, energy	40-Hz response showed no effect of attention. Reduced attention was associated with reduced energy in the 80-120 Hz range with no effects on ITC
Herdman ^90^	Canada	20	Children: 12 Adults: 22	Attention, age	40	Amplitude	Attention significantly enhanced the 40-Hz ASSR in adults only, not children
Skosnik et al.^81^	USA	46	21.35	Chronic cannabis use	10 - 50 in increments of 5	Power, ITC	Reduced 40-Hz power in cannabis users, no differences in ITC
Toso et al.^87^	Germany	20	28	Cortical excitation (via NMDA) and inhibition (via GABA)	40	Total power, PLP	Enhancing GABAergic transmission with lorazepam increased strength of 40-Hz ASSR, blocking NMDA receptors with memantine had no effect on the sustained response
Schuler et al.^75^	Italy	52	29.5	Cortical thickness	40	ITPC, PLV	ITPC and PLV showed significant positive correlation with cortical thickness measured by MRI
Larsen et al.^76^	Australia	45	21.32	Degree of myelination in grey matter	40	PLV, power	Increased myelin content in the right cerebellum was associated with better phase-locking of 40-Hz ASSR. This was specific for PLV and not power
Zhang et al.^88^	China	24	22.96	Emotional arousal	40	PtP amplitude, ERSP	Stronger ASSR in positive emotional state compared to neutral or negative
Melynyte et al.^72^	Lithuania	44	22.5	Gender, handedness	40	PLI, ERSP	Reduced phase-locking and strength of 40-Hz ASSRs in left-handed females compared to right-handed females and left-handed males
Horwitz et al.^89^	Denmark	40	62.02	Intelligence	40 (auditory), 36 (visual)	Gamma power difference between visual and auditory conditions	No significant association between intelligence and auditory response to 40 Hz
Horwitz et al.^94^	Denmark	40	62.02	Long-term memory capacity	40 (auditory), 36 (visual)	Gamma coherence difference between visual and auditory conditions	No significant association between long-term memory and auditory response to 40 Hz
Griškova-Bulanova et al.^73^	Lithuania	28	20.68	Menstrual cycle phase	40	PLI, amplitude, total intensity	PLI was highest in late follicular phase, lowest in mid-luteal phase. Amplitude was largest during late follicular phase, smallest in mid-luteal phase, as was total intensity
Zhang et al.^77^	China	28	20.7	Musical training	40	AEP, PLV	Musicians showed larger steady-state PLVs at high frequencies (40-60 Hz) and lower PLVs at low frequencies (1-20 Hz) compared to non-musicians
Bosnyak et al.^84^	Canada	8	27.5	Musical training	40	Phase, amplitude	Small effect of training on ASSR phase with no significant change to amplitude
Griškova-Bulanova et al.^74^	Lithuania	70	26.07	Processing speed and inhibitory control	30-60 in increments of 1	PLI, ERSP	Faster reaction times correlated with better gamma synchronisation (higher PLI and ERSP values), at 40 Hz and particularly at individual gamma frequencies, no difference for inhibition

a							
Author (Year)	Country	N	Mean Age	Group	Stimulation (Hz)	Outcome Measures	Key Findings
De Stefano et al.^79^	USA	30	13.3	ASD, age	1-100	ITPC, STP	Participants with ASD showed lower ITPC in the 27-39 Hz range than controls in the older but not younger group. ITPC increased with age in typically developing participants but decreased in those with ASD. Adults with ASD showed greater STP in the 20-50 Hz range but no differences were seen in younger group. STP decreased with age in typically developing group but remained constant in ASD group
Seymour et al.^96^	UK	36	16.78	ASD	Auditory and visual, Hz not specified	Power, ITC	Reduced 40-Hz ASSR power and inter-trial coherence in individuals with autism
Granados Barbero et al.^97^	Belgium	54	14.71	Dyslexia	4, 10, 20, 40	SNR, PC	Group with dyslexia showed higher phase coherence and SNR in all frequencies
Lizarazu et al.^95^	France	39	Not reported. Range: 19-40.7	Dyslexia	2, 5, 30	SNR, PLV, IHPS, coherence	Controls demonstrated larger responses to non-speech gamma stimuli in left hemisphere. Failed to replicate other significant group differences from previous research
Wilson et al.^98^	USA	25	42.25	ADHD	40	Amplitude (MEG)	Gamma activity was weaker in ADHD group in the pre-medication, but not the post-medication, condition

a. Overview of neurodevelopmental differences affecting entrainment

For full methodological and task details, see Supplementary Data 1.

For full methodological and task details, see Supplementary Data 2.

*ASD* Autism Spectrum Disorder, *ADHD* Attention-Deficit/Hyperactivity Disorder, *SNR* Signal-to-noise ratio, *PC* Phase coherence, *PLV* Phase-locking value, *IHPS* Inter-hemispheric phase synchronisation, *ITC* Inter-trial coherence, *ITPC* Inter-trial phase consistency, *STP* Single-trial power. All stimulation was auditory unless indicated otherwise in the stimulation column.

*PLV* Phase-locking value, *AMFR* Amplitude modulation following response, *SNR* Signal-to-noise ratio, *PLI* Phase-locking index, *ERSP* Event-related spectral perturbation, *ITPC* Inter-trial phase consistency, *ERF* event-related field, *PtP* amplitude Peak-to-peak amplitude, *IHPS* Inter-hemispheric phase synchronisation, *ITC* Inter-trial coherence, *GFS* Global field synchronisation, *AEP* Auditory-evoked potential, *PLF* Phase-locking factor, *CFC* Cross-frequency coupling, *PLP* Phase-locked power. All stimulation was auditory unless indicated otherwise in the stimulation column.

**Table 2:** Overview of clinical conditions affecting entrainment

Author (Year)	Country	*N*	Mean Age	Clinical Condition	Stimulation (Hz)	Outcome Measures	Key Findings
Chan et al.^102^	USA	43	25.6 (healthy younger group), 64.9 (healthy older group), 75.8 (AD group)	Epilepsy, mild AD	40*	Power	Entrainment was induced across all groups, extending to cortical and subcortical regions distinct from sensory cortex, particularly concentrated in frontal regions for mild AD group
Shahmiri et al.^105^	Iran	42	68.6	AD, MCI	40, 80	ASSR threshold	40-Hz ASSR thresholds were elevated in MCI and AD, respectively, compared to controls. AD group additionally showed higher 80-Hz ASSR threshold compared to controls. The differences between 40- and 80-Hz thresholds decreased with advancing cognitive impairment
van Deursen et al.^101^	Netherlands	55	71.77	MCI, probable AD	40	Power	Power was significantly higher in AD group compared to controls at all electrodes, and significantly higher compared to MCI group at T6. Strong test-retest reliability, moderate correlation between power (T5 and T6) and cognitive performance (ADAS-Cog)
Osipova et al.^103^	Finland	22	71.8	Probable AD	40	Amplitude, power (MEG)	AD patients showed significantly higher amplitude and power compared to healthy controls
Lahijanian et al.^99^	Iran	33	73.18	MCI, mild AD, moderate AD	40	PLV	Improved interregional connectivity and neural synchrony between frontal and parietal regions with strongest effects for MCI patients. Positive correlation between strength of entrainment response and resulting synchronisation
Lahijanian et al.^100^	Iran	11	74.09	Mild AD, non-AD dementia	40	Amplitude, power, PLV, PAC	Required standard of entrainment (according to novel method proposed) was achieved for 4 of 11 dementia patients. Within entrained group, temporal phase stability and spatial phase coupling were maintained. High theta power at rest predicted higher quality of entrainment
Shao et al.^104^	China, USA	11	74.09	Mild AD, non-AD dementia	40	Amplitude	No significant differences between entrained and non-entrained groups on demographic measures (cognitive function assessed by MMSE, age, sex), high theta power at rest predicted higher quality of entrainment
Spydell et al.^106^	USA	26	30	Lesions of midbrain or temporal lobe	40	Phase, RMS power	Phase of 40-Hz response was altered in patients with thalamic or midbrain lesions, but not with lesions of the temporal lobe. Reduced power in patients with midbrain lesions

*For full methodological and task details, see* Supplementary Data 3

*ASSR* Auditory steady-state response, *RMS* Root mean square, *PLV* Phase-locking value, *PAC* Phase-amplitude coupling. *Auditory and visual stimulation.

**Table 3:** Overview of cognitive and biological outcomes of entrainment

Author (Year)	Country	N	Mean Age	Condition/ Trait	Stimulation (Hz)	Stimulation Duration	Outcome Measures	Key Findings
Wang et al.^107^	China	40	22.64	Healthy	40	Single session	Working memory (visuospatial and verbal tasks)	Both accuracy and reaction time improved with 40-Hz stimulation
Chaieb et al.^108^	Germany	25	24.4	Healthy	6, 10, 40	Single session	Long-term memory (word recall), working memory (digit span), vigilance (reaction time)	No significant effects of stimulation on cognitive measures
Engelbregt et al.^109^	Netherlands	24	22.3	Emotionality	40	Single session	Attention (arrow flanker task), working memory (visuospatial task)	Reaction time improved in 40-Hz conditions without increase in errors for attention task, no difference found for working memory. No effect of emotionality observed
Kim et al.^110^	USA	60	24.6	Healthy	18, 40	Single session	Auditory comprehension task performance	No effect of 40-Hz BB on auditory comprehension performance
Hsiung & Hsieh ^111^	Taiwan	30	20.0	Healthy	40*	Single session	Visual threshold and visual spatial memory task performance	No promoting effects of 40-Hz BB on visual threshold or spatial memory tasks observed, reduction of practice effects for visual threshold task only
Manippa et al.^112^	Italy	36	23.3	Healthy	40, 60	Single session	Long-term verbal memory (word recall) and verbal working memory (digit span)	Neither stimulation condition enhanced long-term or working memory, though 60-Hz stimulation reduced intrusion errors
Engelbregt et al.^113^	Netherlands	25	21.8	Healthy	40	Single session	Attention (arrow flanker task reaction time, number of false responses)	Improved performance in BB condition despite lack of evidence for entrainment by EEG
Leistiko et al.^114^	UK	58	26.0	Healthy	40	Single session	Attention network test performance	No effect of 40-Hz BB on attention
Hajós et al.^115^	USA	76	72.0	Mild-moderate AD	40*	1 hour daily for 6 months	Change in MADCOMS, ADCOMS, ADCS-ADL, MMSE, ADAS-Cog 14, CDR, QoL-AD	Active group showed significantly reduced rate of decline on ADCS-ADL and MMSE, and reduced atrophy using MRI. No significant difference between groups for other measures. Mild AE observed
Cimenser et al.^116^	USA	22	70.0	Mild-moderate AD	40*	1 hour daily for 6 months	Functional abilities assessed by ADCS-ADL	Active group maintained functional abilities while control group showed decline. Active group showed reduced nighttime activity while control group showed deterioration in sleep quality. Mostly mild with some moderate and some severe AE
Chan et al.^102^	USA	15	74.4	Mild AD	40*	1 hour daily for 3 months	Cognitive function (assessed by neuropsychological test battery)	Active group showed improved face-name association performance but no other significant differences in cognitive function. Active group showed improved inter-daily stability (actigraphy) and reduced brain atrophy and loss of functional connectivity
He et al.^117^	USA	10	72.0	MCI (prodromal AD)	40*	1 hour daily for 4 or 8 weeks	nan	8-week group showed significant difference in cytokines and immune factors in CSF but no significant changes in Aβ42, t-tau, or p-tau. Functional connectivity between PCC and PCu increased in 8-week group only. Mild AE observed
Da et al.^118^	USA	38	72.49	MCI, AD	40*	1 hour daily for 6 months	nan	Reduced overall and regional white matter atrophy and myelin content loss in active vs. control group with strongest effects in entorhinal region
McNett et al.^119^	USA	11	Not reported	MCI, mild AD	40*	1 hour daily for 6 months	Cognitive function (assessed by MoCA and BoCA)	No statistical comparisons. Most scores improved or remained stable
Da et al.^120^	USA	50	72.14	MCI, AD	40*	1 hour daily for 6 months	nan	Reduced rate of atrophy of corpus callosum in active group compared to sham
Xu et al.^121^	China	4	30.0	Poor sleep quality	40*	1 hour every 5 days for 4 weeks	nan	Improved sleep quality, increased functional connectivity in some brain regions between hippocampus and default-mode network
Liu et al.^122^	China	22	54.11	Insomnia	40*	1 hour daily for 8 weeks	nan	Total sleep time was increased while sleep onset latency and arousal were reduced. Mild AE observed

For full methodological and task details, see Supplementary Data 4.

*BB* Binaural beats, *RCT* Randomised controlled trial, *MRI* Magnetic resonance imaging, *fMRI* Functional magnetic resonance imaging, PET Positron emission tomography, *MADCOMS* Mild-moderate Alzheimer’s Disease Composite Score, *ADCOMS* Alzheimer’s Disease Composite Score, *ADCS-ADL* Alzheimer’s Disease Cooperative Study Activities of Daily Living, *MMSE* Mini Mental State Examination, *ADAS-Cog 14* Alzheimer’s Disease Assessment Scale - Cognitive Subscale-14, *CD*R Clinical Dementia Rating, *CDR-SB* Clinical Dementia Rating – Sum of Boxes, *QoL-AD* Quality of Life in Alzheimer’s Disease, *MoCA* Montreal Cognitive Assessment, *BoCA* Boston Cognitive Assessment, *CSF* Cerebrospinal fluid, *Aβ42* Amyloid beta (42), *T-tau* Total tau, *P-tau* Phosphorylated tau, *PCC* Posterior cingulate cortex, *PCu* Precuneus, *AE* Adverse events, *ARIA* Amyloid-related imaging abnormalities. Safety as an outcome measure denotes adverse events. *Auditory and visual stimulation.

**Table 4:** Overview of individual optimal entrainment frequency

Author (Year)	Country	*N*	Mean Age	Trait	Stimulation (Hz)	Outcome Measures	Key Findings
Aoyagi et al.^129^	Japan	20	3.5 (children), 31.4 (adults)	Age	20–200 in increments of 20	AMFR, SNR	AMFR evoked only by 40 Hz in awake adults, but also at 80 and 100 Hz during sleep. 40-Hz AMFR difficult to detect in children though clearly detected at higher frequencies, especially 80 and 100 Hz (all during sleep).
Poulsen et al.^130^	Canada	60	Timepoint 1: 10 Timepoint 2: 11.5	Age	10 - 100	RMS amplitude, EFR	ASSR amplitude increased with increasing age, as did peak frequency of EFR (35.3 Hz at 10 y to 36.5 Hz at 11.5 y)
Poulsen et al.^131^	Canada	23	29	Age	10 - 100	RMS amplitude, EFR	ASSR amplitude became larger and more stable with age, peak frequency of EFR increased (from 38 Hz at 19 y to 46 Hz at 45 y, overall mean of 41 Hz)
Parciauskaite et al.^123^	Lithuania	37	23.8	Processing speed	35-55 in increments of 1	PLI, ERSP	Group response was maximal between 41-42 Hz with individual peaks ranging from 35–53 Hz, gamma-band responses were negatively correlated with response time for Tower of London task only
Mockevičius et al.^124^	Lithuania	80	26.07	NA	30-60	PLI	Peak individual gamma frequencies were extracted with a mean of 37 Hz in this young adult sample
Artieda et al.^125^	Spain	10	Range: 22-35 years	NA	1-120	ITC, energy	Maximal response was observed around 45 Hz
Pastor et al.^127^	Spain	28	35.3	NA	12, 20, 30, 32, 35, 37.5, 40, 42.5, 45, 47.5, 50, and 60 (EEG) and 12, 32, 40, and 47 (PET)	Amplitude	Amplitude increased in 30–40 Hz range and decreased with stimulation >40 Hz, strongest activation observed by PET at 40 Hz
Zaehle et al.^128^	Germany	21	23.5	NA	20 to 100 in increments of 1	Amplitude	Average ASSR peaked at 48 Hz
Tada et al.^126^	Japan	8	32.3	NA	20, 30, 40, 60, 80, 120, and 160	ITC, ERSP	Both ITC and ERSP were maximal at 40 Hz

For full methodological and task details, see Supplementary Data 5.

*AMFR* Amplitude modulation following response; *SNR* Signal-to-noise ratio; *PLI* Phase-locking index, *ITC* Inter-trial coherence, *ERSP* Event-related spectral perturbation, *RMS* Root mean square, *EFR* Envelope-following-response. *NA* Not applicable.

One additional table (Supplementary Data 6) has been included as part of the supplementary material (S3) to highlight the current clinical trials investigating auditory gamma stimulation as an intervention for neurological conditions with cognitive decline. A final judgement for overall risk of bias as per the appropriate quality assessment tool (AXIS, RoB 2, or ROBINS-I) has been included in each table with the full quality assessments found in the Supplementary Material (S4, Supplementary Data 7, 8, and 9, respectively). Across all included studies, 48 were judged to be at low risk of bias, 12 at moderate risk, and 2 at high risk. The two high-risk studies were characterised by limited information about the participant sample and insufficient statistical reporting; they were included in the tables for completeness but were not used to inform the interpretation of the results. Table of entrainment measures and definitions provided in Table 5.

**Table 5:** Neurophysiological measures and definitions

Measure	Category	Definition	Relevance to entrainment
Phase-locking Index (PLI)^71–74,123,124^, Phase-locking value (PLV)^75–77,95,99,100^	Phase consistency	A measure of the extent to which the instantaneous phase of two signals are synchronised^146^	Measures the degree of neural synchrony to rhythmic stimuli
Inter-trial phase consistency (ITPC)^75,78^, Inter-trial phase coherence (ITPC)^79^, Inter-trial coherence (ITC)^80,81,96,125,126^	Phase consistency	The consistency in phase activity across trials that are time-locked to a specific event^157^	Measures reliability of phase synchronisation to rhythmic stimuli
Phase-locking factor (PLF)^82^	Phase consistency	The degree to which the phases of oscillatory signals align across trials	Quantifies phase consistency in response to rhythmic stimuli
Phase coherence (PC)^97^	Phase consistency	The consistency of the phase differences between two or more brain signals at a given frequency	Measures the degree to which two signals are synchronised in response to rhythmic stimuli
Phase^83,84,106^	Phase consistency	The position within the cycle of a waveform at given points in time	Variability of phase can reflect synchronisation with rhythmic stimuli
Inter-hemispheric phase synchronisation (IHPS)^95^	Phase consistency, coherence (regional)	The consistency of phase relationship between neural oscillations recorded from homologous regions in the left and right hemispheres of the brain^95^	Reflects coordinated neural timing to rhythmic stimuli
Global field synchronisation (GFS)^85^	Phase consistency, coherence (regional)	Estimation of the amount of phase alignment across all brain regions as a function of frequency	Indicates the scale of wide-spread neural coherence in response to rhythmic stimuli
Power^76,81,82,86,87,96,100–103^	Power	The magnitude of brain activity at a specific frequency	Measures the response strength to rhythmic stimuli
Event-related spectral perturbation (ERSP)^72,74,88,123,126^	Power	Changes in the power spectrum of brain activity, specifically the degree of synchronisation or desynchronisation of neural oscillations across different frequencies, evoked by a stimulus^158^	Measures power changes in response to rhythmic stimuli
Phase-locked power (PLP)^87^	Power, phase consistency	The strength of neural activity at a specific frequency that is consistently aligned with a stimulus or event	Reflects the strength of consistent phase alignment to rhythmic stimuli
Root-mean-square (RMS) power^106^	Power	A statistical measure of the strength of a biosignal^159^, in this case, power	Quantifies magnitude of power change in response to rhythmic stimuli
Single-trial power (STP)^79^	Power	The analysis of brain activity based on individual trials of an EEG recording, rather than averaging power across multiple trials	Measures the quantity of neural activity in response to rhythmic stimuli at specific time points
Energy^80,125^	Power	The intensity of a signal simultaneously in time and frequency^160^	Provides an estimation of time-varying magnitude of signal in a frequency band
Power difference between conditions^89^	Power	The difference in signal magnitude associated with one experimental condition to another^89^	Differentiates signal magnitude change between stimulus conditions
Amplitude^71,73,83,84,86,90,93,98,100,103,104,127,128^	Amplitude	The vertical distance or height of a waveform	Measures the response strength to rhythmic stimuli
Amplitude modulation following response (AMFR)^91,129^	Amplitude	A type of auditory-evoked potential that can be recorded from the scalp^161^	Reflects brain response strength to rhythmic stimuli
Event-related field (ERF) amplitude^78,93^	Amplitude	The strength or magnitude of the magnetic field changes in the brain that are elicited by a specific stimulus or event	Measures neural response strength to rhythmic stimuli
Peak-to-peak (PtP) amplitude^88^	Amplitude	The difference between the maximum voltage and the minimum voltage in a cycle, measuring the waveform’s overall variation^88^	Shows neural response magnitude to rhythmic stimuli
Root-mean-square (RMS) amplitude^130,131^	Amplitude	A statistical measure of the strength of a biosignal^159^, in this case, amplitude	Quantifies magnitude of power change in response to rhythmic stimuli
Envelope-following response (EFR)^130,131^	Amplitude	A steady-state evoked response that reflects the auditory processing of a sound’s envelope (fluctuations in amplitude over time)	Reflects the neural tracking of a sound envelope in response to rhythmic stimuli
Total intensity^71,73^	Amplitude	The average amplitude of both phase-locked and non-phase-locked oscillations^73^	Offers a measure of overall increase in signal intensity induced by rhythmic stimuli
Coherence^95^	Coherence (regional)	The synchrony or similarity between two signals	Consistency of neural response to rhythmic stimuli across brain regions
Coherence difference between conditions^94^	Coherence (regional)	The difference in coherence between brain regions associated with one experimental condition to another^94^	Differentiates cross-regional coherence change between stimulus conditions
Cross-frequency coupling (CFC)^82^	Coupling	The interaction between different frequency bands of brain oscillations	Shows the interaction between oscillations in response to rhythmic stimuli
Phase-amplitude coupling (PAC)^100^	Coupling	The interaction where the phase of a slower brainwave modulates the amplitude of a faster brainwave	Shows the coupling between oscillations in response to rhythmic stimuli
Auditory-evoked potential (AEP)^77^	Evoked response	Reflects change in electrical activity of the brain in response to auditory stimuli	Reflects time-locked brain responses to stimuli
Signal-to-noise ratio (SNR)^91,92,95,97,129^	Signal quality	The strength of the desired brain signal relative to unwanted background noise	Measures the quality of the signal in response to rhythmic stimuli
Auditory steady-state response (ASSR) threshold^105^	Response threshold	The minimum sound intensity that elicits a measurable, frequency-specific neural response^105^	Reflects the sound level at which neural activity reliably synchs to rhythmic stimuli
Temporal dynamics^93^	Time-domain analysis	Time-dependent changes and patterns in physiological measures^93^	Reflect time-related changes in response to rhythmic stimuli

Twenty-four of the articles retrieved investigated the effect of different traits on gamma-band entrainment responses (Table 1, Table 1a)^71–94^. Nine of these specifically examined the association between age and entrainment^71,78,79,82,83,86,90,91,93^. While infants did not exhibit a 40-Hz response when measured by AMFR^91^, power at 40 Hz was found to increase linearly from age five and stabilise in early adulthood, with concomitant increases in amplitude variability^86^. Another study reports that power, PLF, and CFC increased from ages eight to 16 years before decreasing between 20 and 22^82^. A linear decline in evoked amplitude and PLI with age was reported, though their total intensity measure remained unaffected^71^. ITPC was found to increase with age^78,79^, while ERF^78^ and STP decreased^79^. Contextual factors moderated the effects of age, whereby quiet conditions increased 40-Hz amplitude in younger adults compared to older adults^93^, while attention significantly enhanced 40-Hz amplitude in older adults but not children^90^. One study found no effects of age on entrainment^83^.

Other traits identified included processing speed, where reaction time (RT) was negatively correlated with PLI and ERSP at individual optimal gamma entrainment frequencies and at 40 Hz, indicating that subjects with better gamma synchronisation were faster to execute behavioural responses^74^. Attention appeared to enhance the entrainment response, as SNR improved when participants were instructed to attend to the 40-Hz stimuli^92^, while GFS weakened when participants were distracted from the auditory input^85^. However, one study found no effect of attention on ITC or energy^80^.

Neuroanatomical differences were also linked to variations in entrainment, with increased cortical thickness associated with increased PLV and ITPC^75^, and myelination in the right cerebellum corresponding to increased PLV^76^. Long-term lifestyle factors were also associated with variability, such as musical training, which showed some association with larger PLVs at 40 Hz^77^, and shortening of ASSR phase, but no change in amplitude^84^, and chronic cannabis use, which was associated with reduced gamma power^81^.

Stable traits such as handedness and sex revealed reduced PLI and ERSP of 40 Hz in left-handed females compared to right- and left-handed males^72^. Variable physiological states, such as the current phase of the menstrual cycle^73^ and GABAergic neuronal inhibition^87^ affected PLI and amplitude, and total power, of the gamma-band response, respectively. Moreover, emotional arousal, induced via emotional video clips, affected entrainment response, with stronger ASSRs found (measured by PtP and ERSP) in positive compared to neutral or negative emotional states^88^.

Importantly, of all traits studied, none were found to affect the neural response to the extent that entrainment could not be achieved, with the exception of age in very young children, though this was only observed when measured by AMFR.

Five studies explored how neurodevelopmental conditions, including ASD, ADHD, and dyslexia, affect entrainment^79,95–98^. Higher phase coherence and SNR were reported in individuals with dyslexia^97^, however, another study found minimal differences between dyslexic and control groups when assessing SNR, PLV, IHPS, and coherence^95^. Adults with ASD demonstrated reduced 40-Hz power and ITC^96^. Older adults with ASD also showed reduced ITPC, but this was not found in younger adults with ASD^79^. Those with ADHD showed reduced amplitude with 40-Hz stimulation only in the pre-medication condition and not after receiving their daily stimulant medication^98^.

Of the seven articles retrieved examining the effect of clinical conditions on the entrainment response, all but one focused on dementia, including MCI, AD, and non-AD dementia^99–105^. The remaining study investigated the entrainment response, as measured by phase and RMS power, among patients with lesions affecting the midbrain or temporal lobe^106^. All studies were successful in inducing gamma entrainment in dementia patients to some degree, as determined by ASSR threshold, power, amplitude, PLV, and PAC. ASSR thresholds^105^, power^101,103^, and amplitude^103^ were significantly higher in AD compared to controls, with ASSR thresholds and power also significantly elevated in AD patients relative to MCI^101,105^. Improved neural synchronisation and connectivity in MCI and AD patients, indexed by PLV, between intraregional and interregional sites were found, with the strongest effects shown in MCI patients^99^. Interestingly, one feasibility study^102^ found that the entrainment response was spread across multiple areas in a cognitively normal participant group but concentrated around frontal regions in mild AD (measured by PSD and coherence), possibly reflecting disease-related changes in sensory stimulation response.

However, Lahijanian et al.^100^ claimed that a sufficient standard of entrainment was achieved for only a subset of their study’s participants, based on their proposed definition of the ‘entrained’ brain; a peak frequency amplitude response at the stimulation frequency at least three standard deviations above the mean amplitude response in a range of adjacent frequencies. The authors additionally found that higher theta power (4 – 8 Hz) at rest predicted quality of entrainment in dementia patients, indexed by an averaged z-score value of the 40-Hz component’s amplitude, an example of an individual difference influencing the neural response within a clinical population.

Seventeen papers of those retrieved outlined downstream cognitive and biological effects of auditory gamma stimulation^102,107–122^. Eight of these were single-stimulation session studies examining cognitive effects in healthy individuals, reporting mixed findings on cognitive outcomes^107–114^. Forty-hertz binaural beat (BB) stimulation improved RT without increasing errors on an attention task^109^, enhanced both accuracy and RT on visuospatial and verbal working memory tasks^107^, and reduced false responses on the flanker task (even when there was no evidence of entrainment as indexed by absolute power)^113^. Further, 60-Hz BB stimulation reduced intrusion errors on word recall and digit span tasks^112^. Conversely, some studies found no effects on cognitive performance including word recall accuracy, digit span accuracy, RT^108^, auditory comprehension accuracy^110^, visual threshold, and visual spatial memory task accuracy^111^, or the error rate and RT of an attention network task^114^.

Nine studies retrieved employed chronic entrainment (one-hour daily sessions over several weeks or months), demonstrating some improvements across cognitive and biological outcomes^102,115–122^. These included maintenance or enhancement of functional abilities and cognitive scores (assessed by ADCS-ADL, MMSE, face-name association task, MoCA, BoCA)^102,115,116,119^, reductions in brain atrophy and white matter loss observed using MRI^102,115,118,120^, enhanced functional connectivity measured by fMRI^102,117^, and improved sleep quality and daily rhythmicity^102,116^. In cognitively healthy participants with either sleep problems or a diagnosis of insomnia, interventions were associated with improved sleep quality and increased functional connectivity (fMRI), particularly within the hippocampus and default-mode network^121,122^.

The outcomes of chronic gamma stimulation appeared to vary across disease stages of AD. For individuals with MCI, a reduction in overall and regional white matter atrophy, decreased myelin content loss, particularly in the entorhinal region, and slower rates of atrophy in the corpus callosum were observed with daily gamma stimulation sessions over a six-month period^118,120^. Further, increased functional connectivity between the posterior cingulate cortex and precuneus was reported in patients with MCI, when daily stimulation was applied for only eight weeks^117^. Improved performance on a face-name association task, reduced brain atrophy and loss of functional connectivity were observed in mild AD when stimulation was applied daily for three-months^102^. One study reported stability in outcomes with no significant improvements or declines in cognitive or biological measures in MCI or mild AD following daily stimulation for six months^119^. While fewer significant results were observed for moderate AD, some studies found a reduced rate of decline in cognitive measures and functional abilities, and reduced night-time activity following daily stimulation for six months^115,116^. No studies observed a significant difference in Aβ levels (measured by amyloid-PET and cerebrospinal fluid).

Mild adverse events (AE) were commonly reported across studies^115–117,121,122^, including headache, dizziness, and tinnitus. One moderate (chest irritation) and one severe (dementia exacerbation) AE were also reported^116^.

Nine studies of those retrieved used a range of stimulation frequencies to determine the optimal frequency for inducing entrainment in the given sample, according to different attributes of the brain response^123–131^. The stimulation frequency producing maximal gamma entrainment varied considerably between studies of adults alone, from 37 Hz^124^ to 48 Hz^128^ measured by PLI and EFR respectively. In children, one study indicated that the optimal entrainment frequency is around 36 Hz measured by EFR^130^. Among younger adults (19-35 years), optimal entrainment frequencies as determined by different measures were 37 Hz (PLI)^124^, 38 Hz (EFR, RMS amplitude)^131^, 40 Hz (amplitude)^127^, 40 Hz (AMFR, SNR)^129^, 40 Hz (ITC, ERSP)^126^, 41.5 Hz (PLI, ERSP)^123^, 45 Hz (ITC, energy)^125^, and 48 Hz (amplitude)^128^. In adults up to age 45, one study indicated that the strongest response was observed with stimulation at 46 Hz (EFR, RMS amplitude)^131^. No studies investigated optimal entrainment frequency in adults older than 45.

This search retrieved 14 clinical trial registry entries from ClinicalTrials.gov and the WHO ICTRP, 10 of which are ongoing (See Supplementary Materials S3, Supplementary Data 6). Where results of a clinical trial have been published, the corresponding reference has been included in the final column of Supplementary Data 6.

**Fig. 2:**
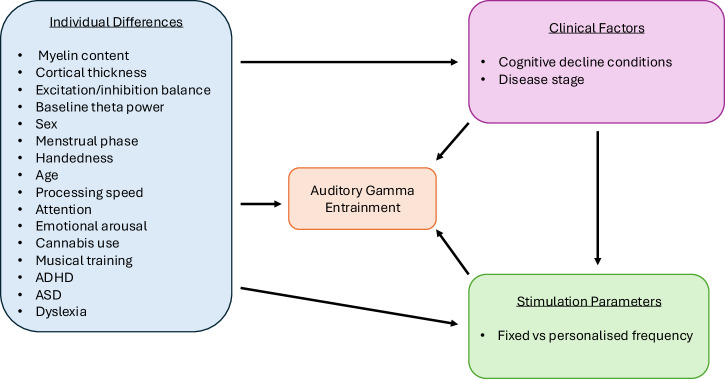
Schematic representation of the factors influencing auditory gamma entrainment. Individual differences (blue) (biological, cognitive, and lifestyle traits) and clinical factors (pink) (conditions involving cognitive decline and disease stage) both directly affect entrainment outcomes (orange). Individual differences may also influence the severity of clinical outcomes, which in turn further shape entrainment responses. Moreover, some individual and clinical factors may shift the gamma frequency at which entrainment is most effective, highlighting the potential need for personalised stimulation approaches (green). Together, these domains underscore the multifactorial nature of variability in auditory gamma entrainment.

To synthesise the findings, we developed a schematic model (Fig. 2) illustrating the key factors identified in the review as influencing auditory gamma entrainment.

## Discussion

This systematic review examined responses to and effects of auditory (and audiovisual) gamma stimulation across healthy and clinical populations, identifying 16 distinct characteristics that appear to influence entrainment across studies. We aimed to elucidate how individual differences and clinical conditions may affect gamma entrainment and its associated cognitive and biological outcomes. Our findings suggest several promising patterns in the data, though the field would benefit from improved methodological standardisation to strengthen future research, particularly with respect to entrainment measurement in clinical populations. Below, we summarise the main findings and implications of the review.

A principal challenge encountered in this review is the considerable heterogeneity in methods used to measure entrainment, notably with techniques developing over time, reflecting a fragmented understanding of ‘effective’ entrainment. Our analysis revealed 32 different terms used to quantify entrainment, many of which overlap in methodological terms (see Table 5). These metrics fall into several broad categories: phase consistency, amplitude, power, coupling, coherence, and event-related responses.

The divergence in methodological approaches poses a significant barrier to cross-study comparisons, as the chosen quantification method may influence the interpretation of neural entrainment outcomes. As a result of this methodological heterogeneity, it becomes challenging to determine whether observed differences represent genuine variations in neural synchronisation or artefacts of the measurement technique employed. This underscores the need to work toward a consensus on entrainment metrics in future research.

The lack of consensus in entrainment metrics extends beyond academic discourse; it also has significant implications for the clinical translation of sensory gamma stimulation interventions. In several studies targeting populations with MCI and AD, elevated gamma power and amplitude were reported^101,103,105^. However, the mere presence of power in a given frequency band does not necessarily imply the presence of an oscillation at that frequency, as evoked potentials or muscle and eye-movement artefacts, for example, will yield power in multiple frequency bands even in the absence of oscillations^132^. Importantly, no study investigating entrainment outcomes in clinical cohorts in this review looked at metrics of phase. As such, studies evaluating entrainment efficacy in clinical cohorts relying solely on power and amplitude metrics may potentially overlook critical aspects of oscillatory dynamics that may be essential for accurately capturing entrainment. For instance, a study using phase-locking indices might detect significant entrainment where another using power-based measures might not, even when analysing the same underlying neural data.

This issue becomes especially pertinent in clinical applications. Reliance on a single entrainment measure, such as amplitude or power, risks excluding patients who may demonstrate entrainment via alternative indices, such as phase synchronisation. Indeed, while some studies have excluded ‘non-entrained’ participants based solely on a 40-Hz-specific amplitude criterion^100^, others have documented cognitive benefits even in the absence of significant power increases^113^, indicating that entrainment might not be fully captured by power metrics alone. If participants are to be excluded on the basis of ‘entrainability’, it is essential that the operational definition and measurement of entrainment are rigorously validated. These discrepancies highlight the importance of moving toward a unified, consensus-based approach to measuring entrainment – one that more comprehensively captures its dynamics in clinical populations and enhances translational insights into neurocognitive function. This is especially important considering the growing number of clinical trials employing gamma stimulation in clinical populations, as evidenced by this review (S3).

Nonetheless, important individual differences in auditory gamma entrainment were found. Of the 16 differences identified by our search as influencing the entrainment response, eight were biological in nature, including myelin content, cortical thickness, the excitation/inhibition balance, baseline theta power, sex, menstrual phase, handedness, and age. Cognitive performance measures - indexing processing speed, attention, and emotional arousal - as well as lifestyle factors such as cannabis use and musical training, appear to further modulate entrainment. In addition, neurodevelopmental conditions such as ADHD, ASD, and dyslexia have been associated with altered patterns of auditory gamma entrainment.

Although no single trait consistently predicted individual differences in auditory gamma entrainment, age emerged as one of the most frequently examined characteristics, which is particularly relevant for interventions in cognitive decline. However, the wide range of entrainment metrics used across studies made it difficult to compare studies directly. Some studies reported increased oscillatory power with age; for example, one study observed a linear increase from childhood through middle adulthood^86^. Given evidence of age-related changes in GABAergic function^133,134^, which is responsible for the generation and modulation of gamma oscillations^135,136^, increased gamma power observed with advancing age may reflect changes in inhibitory neural circuits^137^. However, the concurrent decline in PLI found in 20- to 58-year-olds^71^ could indicate reduced precision in neural synchrony. It could be that while the overall signal intensity may be preserved or even elevated, the fine-tuning of neural timing may become less effective with age. Notably, older adults were underrepresented in the studies reviewed. Nonetheless, these patterns underscore the need to distinguish between entrainment metrics; while higher power is often interpreted as indicative of stronger entrainment, in ageing populations, it may instead reflect compensatory activity or normal age-related shifts in cerebral network function^138^.

Understanding these inter-individual differences is likely to be important for optimising auditory gamma stimulation as a therapeutic tool. If future research can clarify their influence on the neural response, it may become feasible to adapt stimulation protocols to individual profiles, thereby maximising therapeutic outcomes. However, until the field reaches consensus on how entrainment is measured, drawing firm conclusions about the role of these individual differences will remain challenging.

Importantly, although 40 Hz remains the most commonly used frequency for auditory gamma stimulation, evidence from this review suggests that the optimal frequency for entrainment may vary between individuals. Several studies report stronger entrainment responses at frequencies ranging from 37 to 48 Hz^123–125,128,131^ across different age groups. Age-related variation appears particularly relevant, with some findings indicating that older adults respond more effectively to lower gamma frequencies^139^, an effect that may be especially important in dementia. In support of this, reduced GABA levels in ageing have been linked to lower peak gamma frequencies for entrainment^140^. It has also been proposed that the optimal entrainment frequency for humans may differ from that in animal models^26^. These findings raise questions about the widespread reliance on 40 Hz and suggest that fixed-frequency stimulation protocols may not fully capture individual variability in neural dynamics, which could influence efficacy.

Compounding this issue is the diversity of metrics used to determine optimal entrainment frequency. Consequently, the optimal frequency for entrainment identified using one measure may not correspond to that determined by another, leading to inconsistencies across studies. Moreover, while preliminary evidence from recent studies suggests a possible relationship between real-time 40-Hz EEG activity and subsequent cognitive improvements^69^, it remains unclear whether stimulating at a theoretically ‘optimal’ entrainment frequency would in fact confer greater therapeutic benefits. Establishing a potential link between entrainment strength and clinical outcomes, therefore, represents a key objective for future investigations.

The review further highlights a complex relationship between entrainment and the neurobiological substrates of cognitive function – a relationship which remains underexplored. In clinical populations, namely MCI or AD, increased gamma power and amplitude have been associated with cognitive decline^101,103,105^. Such increases may reflect compensatory mechanisms or a state of neuronal hyperexcitability in AD^141–143^. It is possible that measures of phase synchronisation could offer a particularly sensitive index of entrainment and may prove more sensitive to disease progression, thereby providing additional mechanistic insight. In this review, a positive correlation was observed between cortical thickness and PLV and ITPC in healthy participants^75^. Accordingly, these phase-based measures may be expected to decline with the brain atrophy characteristic of AD, in which cortical thickness is reduced^144^. However, none of the reviewed studies incorporated phase measures in clinical cohorts. Beyond cognitive function, our review also found little investigation of disease-specific factors that might shape the response to stimulation, despite indications that therapeutic benefits may depend on the stage of neurodegeneration^145^. Neural circuits may exhibit progressively attenuated responses to external stimulation with increasing disease severity, in a manner less readily captured by power measures, suggesting a need to characterise responsiveness across the course of neurodegeneration, to identify stages of diminishing neuroprotective effects.

In extension of this concern, where dementia patients are excluded for not meeting amplitude-based criteria for entrainment^100^, there is typically little consideration given to possible explanations for non-entrainment. This selective exclusion ensures the attributes or processes underlying subthreshold or absent entrainment, as determined by a singular metric, will remain understudied. Given the growing number of clinical trials in cognitive decline and auditory gamma stimulation, some of which require EEG evidence of entrainment for inclusion, it is increasingly important to develop a better understanding of the neurophysiological determinants of entrainment to expand its clinical utility.

Nevertheless, emerging evidence from cognitive and biological outcomes suggests that sensory gamma entrainment may have the potential to influence aspects of cognitive decline, with reported improvements in daily functioning, sleep quality, brain atrophy, connectivity, and cognitive performance. However, these benefits are not uniformly reported, and no study has yet demonstrated a significant change in amyloid levels – a finding that contrasts with preclinical rodent work. This discrepancy may reflect species-specific neurobiology or differences in stimulation parameters, such as duration. Chronic stimulation appears more effective than single-session exposure, and the reviewed studies indicate that longer-term stimulation may be necessary for robust cognitive and biological effects. Collectively, these findings point to the therapeutic promise of auditory gamma stimulation while exposing the current methodological narrowness: a broader range of strategies for both inducing gamma entrainment and quantifying its neurophysiological impact must be explored to realise its full potential.

It is also important to note that most long-term clinical studies in this review reported mild AE such as headaches, dizziness, and tinnitus^115–117,122^. Future research should prioritise the development of less obtrusive sensory gamma stimulation methods, particularly for vulnerable populations who require prolonged treatment, such as individuals with AD, for whom exposure to intense visual and auditory stimuli potentially limits feasibility and long-term adherence to stimulation-based interventions.

While this systematic review provides an overarching view of existing research on auditory gamma entrainment, certain limitations must be acknowledged. First, there were minor discrepancies between the registered PROSPERO protocol and the final review. Specifically, the search strategy was expanded to include manual and AI-assisted searches, the inclusion criteria were broadened to encompass grey literature and neurodevelopmental conditions, and risk of bias assessment tools were adapted post hoc. These modifications were made to enhance completeness and methodological rigour, but they represent deviations from the original protocol and should be considered when interpreting the review. Second, the use of AI-assisted tools to support the literature search and broaden coverage remains an emerging approach that has not yet been standardised, which may limit reproducibility and hinder exact replication by other researchers. Third, the search was restricted to English and French publications, which may have introduced a degree of selection bias by excluding potentially relevant studies published in other languages. Beyond these methodological issues, the included studies varied considerably in design, sample size, stimulation protocols, outcome measures, and definitions of effective entrainment, which made synthesis challenging. The broad range of entrainment measures further complicates comparability, as does the difficulty of separating associations from experimental conditions, especially where multiple traits were studied simultaneously. Some ambiguity also emerged in distinguishing between stable traits and more context-dependent influences – such as attention – within the variables reviewed. For consistency, we included only those factors generalisable to intrinsic individual differences, although the contribution of external influences, such as stimulation parameters or contextual factors, remains important for future investigation.

We suggest that future research would benefit from prioritising the use of PLI as the primary metric for assessing auditory gamma entrainment. Phase-based measures are arguably the most direct measure of entrainment, defined as alignment of oscillations to an external rhythm^20^. PLI offers several advantages in comparison to other measures: it directly quantifies the temporal alignment of neural responses to external stimuli, is less susceptible to confounding from amplitude fluctuations, and may be more robust to noise^80,146–148^. Phase-based measures may also demonstrate greater reliability than power-based metrics^149^. Other indices, such as power or coherence, may provide complementary information on signal magnitude and network-level interactions, or even be more appropriate for certain research questions. Nonetheless, because phase synchronisation most directly captures the temporal alignment of neural responses, we propose that it serves as the principal, albeit not exclusive, metric for assessing entrainment.

Adopting a unified metric of entrainment would not only facilitate more rigorous cross-study comparisons but also enhance the translational potential of auditory gamma stimulation and entrainment interventions more broadly. With a consistent approach to measurement, it would become feasible to conduct meta-analyses capable of identifying robust predictors of therapeutic response and informing the development of tailored intervention strategies. Establishing such a consensus will be essential if auditory gamma stimulation is to progress from a promising experimental approach to a potentially clinically reliable intervention.

Several critical gaps in the literature warrant further investigation. First, there is a pressing need to determine whether a causal relationship exists between the degree of entrainment and the cognitive and biological outcomes reported in both healthy and cognitively impaired populations. Second, the field must address if the efficacy of auditory gamma stimulation declines as neurodegenerative disease progresses. Clarifying the pattern of stage-dependent efficacy will be essential for informing early intervention strategies and identifying patient populations most likely to benefit from gamma stimulation therapies. Third, systematic exploration of individualised stimulation protocols - including frequency tuning - is warranted. As evidence accumulates that optimal entrainment parameters may vary between individuals, future studies should evaluate the comparative benefits of personalised versus standard stimulation approaches. Demonstrating the feasibility and efficacy of tailored protocols would represent a significant advancement in the clinical application of auditory gamma stimulation.

Finally, there is a need to investigate the long-term effects of chronic auditory gamma stimulation. Extended treatment durations may be required to elicit demonstrable cognitive and neurobiological benefits observed in preclinical models, which have not yet been replicated in human studies. However, long-term studies must also carefully consider the comfort and safety of sustained stimulation, particularly for vulnerable populations. Future research should support the development of more tolerable stimulation methods to improve feasibility and adherence.

In summary, this systematic review has highlighted both the promise of and challenges endemic to the field of auditory gamma stimulation. The evidence indicates that individual differences, spanning biological, cognitive, lifestyle, and neurodevelopmental factors, play a role in shaping neural responses to auditory stimulation. Moreover, while preliminary findings suggest that auditory gamma stimulation may confer cognitive and biological benefits in neurological conditions with associated cognitive decline, the heterogeneity in measurement techniques and stimulation protocols complicates the interpretation of these findings.

To advance the field, we recommend the following:

1. Consensus on entrainment metrics: Prioritise the use of PLI as the core metric of auditory gamma entrainment, supplemented by complementary measures, such as power or coherence.
2. Causal investigations: Design experimental studies that manipulate entrainment parameters to determine whether the degree of neural synchronisation causally influences the observed cognitive/biological outcomes.
3. Personalised protocols: Evaluate the benefits of individualised stimulation frequencies and tailored intervention strategies in comparison to standardised protocols.
4. Early intervention focus: Identify patterns of disease progression associated with attenuated responsiveness to auditory gamma stimulation, thereby informing patient selection and optimal timing of intervention.
5. Long-term studies: Conduct longitudinal research to evaluate the effects of chronic stimulation on both efficacy and tolerability, particularly in vulnerable populations.

By addressing these priorities, future research can overcome the limitations imposed by methodological heterogeneity and advance the clinical translation of sensory gamma stimulation into a highly impactful intervention for cognitive decline. In doing so, the field will not only deepen our understanding of neural synchronisation mechanisms but also contribute to the development of personalised, evidence-based treatment strategies for neurological disorders.

## Methods

The systematic literature review was conducted according to the Preferred Reporting Items for Systematic Reviews and Meta-Analyses (PRISMA) 2020 guidelines^150^ and the study’s pre-registered protocol on PROSPERO: CRD42024590002.

### Search strategy

A systematic literature search was conducted on 1/10/2024 (updated on 26/11/2024) using the following electronic databases on the Ovid platform: MEDLINE, Embase, and APA PsycInfo. Additional searches were performed on ClinicalTrials.gov and the World Health Organisation International Clinical Trials Registry Platform (WHO ICTRP) to reduce evidence selection bias^151^. Databases were searched from their inception up until November 2024. An example of the terms and combinations applied to search within titles, abstracts, and/or keywords in the relevant databases is as follows: (gamma or “high-frequency” or “high frequency” or “40 Hz” or 40 Hz or “40-Hz” or “gamma-band” or “gamma band”). The full list of search terms and combinations for each database is provided in Supplementary Materials (S1). The search was restricted to studies conducted with human subjects and available in English or French.

### Eligibility criteria

The review focused on studies utilising gamma-frequency auditory stimulation for the entrainment of gamma oscillations, whether applied independently or in conjunction with visual stimulation. Studies were included if they involved healthy individuals or individuals diagnosed with neurological conditions associated with cognitive decline, such as AD or MCI. Eligible studies were required to report EEG measures of entrainment, cognitive outcomes, or biological changes. Additionally, animal studies were excluded to align the review’s scope with the focus on human populations and translational applicability. Grey literature (non-peer-reviewed sources), including clinical trial registry entries and journal articles in preprint, were included in the records maintained, so as to minimise publication bias and provide an overview of ongoing research in the rapidly growing field^152^.

### Data selection

In accordance with the PRISMA 2020 guidelines^150,153^, two authors (EB and ADB) independently screened all titles and abstracts retrieved from the database search. The selected papers were cross-checked to confirm agreement, with any discrepancies resolved by a third author (GM). Full texts of the remaining articles were assessed against the predefined inclusion and exclusion criteria to determine their eligibility for review. A manual reference list search of 10 highly relevant papers was then conducted, and artificial intelligence (AI) tools were employed to identify studies that may have been missed by the initial search (See S2 for the detailed list).

### Data extraction and analysis

Data were extracted by authors EB and ADB from studies meeting the inclusion criteria and deemed eligible for review. The following information was retrieved: author(s), publication date, country, study design, population, sample size, percentage female, mean age, trait (or clinical condition(s) for clinical studies), stimulation modality, stimulation frequency, experimental paradigm (conditions and/or task), comparison group, outcome measures (measures of entrainment or cognitive/biological effects including neurobiological and physiological responses), and main findings of relevance. Data extraction was recorded using Microsoft Excel (Version 16.66.1).

### Risk of bias

Two authors (EB and ADB) independently evaluated the risk of bias and quality for each study. For observational studies, the appraisal tool for cross-sectional studies (AXIS)^154^ (Supplementary Data 7) was employed, while the Cochrane Risk of Bias 2 (RoB 2)^155^ (Supplementary Data 8) or Risk Of Bias In Non-randomised Studies - of Intervention (ROBINS-I)^156^ (Supplementary Data 9) tools were used for randomised controlled trials (RCT) or non-randomised trials of interventions respectively. Discrepancies in quality assessments were resolved through consensus discussion between the authors.

## Supplementary information

Supplementary Material

Supplementary Data 1

Supplementary Data 2

Supplementary Data 3

Supplementary Data 4

Supplementary Data 5

Supplementary Data 6

Supplementary Data 7

Supplementary Data 8

Supplementary Data 9

## Data Availability

All data generated or analysed during this study are included in this published article and its supplementary information files.
