# Supplementary material for: Efficacy of auditory gamma stimulation for cognitive decline: a systematic review of individual and group differences across cognitively impaired and healthy populations
Source: NPJ Aging. 2025 Dec 5;12(1):8. doi: 10.1038/s41514-025-00305-1 (PMC12795826; doi:10.1038/s41514-025-00305-1)
Supplement: Supplementary file 1 — Supplementary Material [file 41514_2025_305_MOESM1_ESM.pdf]

Contents

S1: Search terms **.......... 2**

S2: Manual and Artificial Intelligence searches **.......... 6**

S3: Clinical trials **.......... 8**

Supplementary Data 6. Clinical trials using auditory gamma entrainment for cognitive decline in progress.......... 9

S4: Quality assessment **.......... 10**

Supplementary Data 7. Risk of bias assessment for cross-sectional studies. 10

Supplementary Data 8. Risk of bias assessment for randomised controlled trials. 10

Supplementary Data 9. Risk of bias assessment or non-randomised trials. 10

S5. PRISMA 2020 Checklist **.......... 11**

References **.......... 14**

S1: Search terms

OVID

MEDLINE, Embase, and APA PsycInfo were searched simultaneously using the Ovid platform. Limits were then imposed to limit the resulting search to human studies available in English or French only. Duplicate results were then removed from the multi-database search using the Ovid de-duplication tool with 'Database Preferences' settings favouring MEDLINE (1), Embase (2), and APA PsycInfo (3) respectively. No field preferences were specified for the de-duplication to retrieve the most comprehensive search results possible. The following search terms were used in accordance with Ovid guidelines:

(("individual differences" or differen* or "inter-individual" or varia* or heterogeneity or predict* or optim* or precision or individuali* or personalis* or personaliz* or tailor* or respons* or factor* or sensitiv* or sex or age or older or younger or male* or female* or gender or elderly or healthy or clinical* or neurological or neurodegenerat* or "cogniti* impair*" or "cogniti* declin*" or "cogniti* defici*" or "cognitive dysfunction" or dementia or Alzheimer* or "memory loss" or "memory impairment") and (gamma or "high-frequency" or "high frequency" or "40 Hz" or 40Hz or "40-Hz" or "gamma-band" or "gamma band") and ((auditor*y or sensor*y or audiovisual or sound or rhythm* or aural or audio or "click train*") adj3 (evok* or exogenous or stimulat* or modulat* or external* or induc* or generat* or modif* or neuromodulat* or entrain*)) and (entrain* or synchron* or oscillat* or cortical or activ* or wave* or brainwave* or power or coupling or modulat* or rhythm* or coheren* or neurophysiolog* or electrophysiolog* or electroencephalograph* or eeg or magnetoencephalograph* or meg or respons* or potential* or erp or "steady-state" or assr or modif* or cognitive or task or "cognitive performance" or "cognitive function" or memory

or attention or learning or processing or executive or "problem-solving" or "problem solving" or behavio* or assessment or control or "cognitive rehabilitation" or evaluation or outcome or improv or intell* or neuropsychological* or brain or neural or neuronal or atrophy or synap* or patholog* or amyloid* or tau or inflammat* or neuroinflammat* or neurofi* or plaque* or accumulat* or cerebrospinal or csf or biomarker* or "white matter" or "gr*y matter" or pet or mri or aggregat* or protein* or neuroprotecti* or "myelin* neur* circuit*" or neurocircuit* or "neur* network*" or connectivity or neurotoxic* or restor* or therap*)).tw. not (tdcs or "Transcranial direct current stimulation" or tacs or "Transcranial alternating current stimulation" or tes or "Transcranial electrical stimulation" or dbs or "Deep brain stimulation" or "Peripheral nerve stimulation" or tms or "Transcranial magnetic stimulation" or rtms or brainstem or "Spinal cord stimulation" or tinnitus or "Fragile X" or rat* or mouse or mice or rodent* or theta or beta or alpha or delta or "low-frequency" or review or macaque or audiolog* or bipolar or schizophrenia or "post-traumatic stress disorder" or motor or "deep brain modulation" or gait or walking or spinal or diabet* or nest* or animal or cross-species or non-human or pain).ti.

<u>ClinicalTrials.gov</u>

Condition/disease:

("cognitive" OR "cognition" OR "cognitively" OR "decline" OR "dysfunction" OR "dementia" OR "Alzheimer's disease" OR "Alzheimer disease" OR "Mild Cognitive Impairment" OR "Mild Cognitive Disorder" OR "MCI" OR "neurodegenerative" OR "neurodegeneration" OR "degeneration" OR "atrophy" OR "neurological" OR "memory")

Intervention/treatment:

("auditory" OR "auditorily" OR "sensory" OR "sensorily" OR "sound" OR "click" OR "audio" OR "audiovisual" OR "rhythmic" OR "rhythm" OR "aural")

AND

("entrainment" OR "stimulation" OR "stimulation" OR "neuromodulation" OR "neuromodulator" OR "neuromodulators" OR "evoked" OR "modulating" OR "modulated" OR "modulation")

Other terms:

("gamma" OR "gamma-band" OR "high frequency" OR "high-frequency" OR "40 Hz" OR "40Hz" OR "40-Hz")

Title:

NOT ("transcranial" OR "transcutaneous" OR "electrical stimulation" OR "deep brain stimulation" OR "magnetic stimulation")

<u>WHO International Clinical Trial Registry Platform</u>

Title:

("cogniti*" OR "declin*" OR "dysfunction" OR "dementia" OR "Alzheimer's disease" OR "Alzheimer disease" OR "Mild Cognitive Impairment" OR "Mild Cognitive Disorder" OR "MCI" OR "neurodegenerati*" OR "degeneration" OR "atrophy" OR "neurological" OR "memory") AND ("gamma" OR "gamma-band" OR "high frequency" OR "high-frequency" OR "40 Hz" OR "40Hz" OR "40-Hz") AND ("auditory" OR "auditorily" OR "sensory" OR "sensorily" OR "sound" OR "click" OR "audio" OR "audiovisual" OR "rhythm*" OR "aural") AND ("entrainment" OR "stimulation" OR "neuromodulation" OR "neuromodulator*" OR

"evoked" OR "modulating" OR "modulated" OR "modulation") NOT ("transcranial" OR "transcutaneous" OR "electrical stimulation" OR "deep brain stimulation" OR "magnetic stimulation")

With 'Recruitment status' set to 'ALL'.

S2: Manual and Artificial Intelligence searches

Manual reference list search

The following 10 papers were hand-picked as those deemed most relevant to the research questions, for manual searching of the reference list:

- Gamma sensory entrainment for cognitive improvement in neurodegenerative diseases: opportunities and challenges ahead[1]
- Gamma Oscillations in Alzheimer's Disease and Their Potential Therapeutic Role[2]
- Neuronal Network Oscillations in Neurodegenerative Diseases[3]
- Gamma oscillations in cognitive disorders[4]
- Modulating Gamma Oscillations Promotes Brain Connectivity to Improve Cognitive Impairment[5]
- Gamma Entrainment: Impact on Neurocircuits, Glia, and Therapeutic Opportunities[6]
- A review of gamma oscillations in healthy subjects and in cognitive impairment[7]
- Optimal flickering light stimulation for entraining gamma waves in the human brain[8]
- Optimal flickering light stimulation for entraining gamma rhythms in older adults[9]
- An update on the use of gamma (multi)sensory stimulation for Alzheimer's disease treatment[10]

AI tools used to find studies potentially missed by search terms

The following papers were fed into the *ConnectedPapers* tool to identify related publications:

- Gamma sensory entrainment for cognitive improvement in neurodegenerative diseases: opportunities and challenges ahead[1]
- Gamma oscillations in cognitive disorders[4]
- Gamma-Range Auditory Steady-State Responses and Cognitive Performance: A Systematic Review[11]
- Safety, tolerability, and efficacy estimate of evoked gamma oscillation in mild to moderate Alzheimer's disease[12]
- An update on the use of gamma (multi)sensory stimulation for Alzheimer's disease treatment[13]
- Individualized EEG-Based Neurofeedback Targeting Auditory Steady-State Responses: A Proof-of-Concept Study[14]

The following prompts were fed into both the *Elicit* and *Perplexity* search tools to identify relevant publications:

- How do individual characteristics, such as age or sex, influence the effectiveness of auditory gamma stimulation for entrainment in the healthy brain as well as any resulting effects on cognition?
- How do the effectiveness and cognitive outcomes of auditory gamma entrainment differ across neurological conditions which may impair cognitive function compared to healthy populations?
- How might individual characteristics interact with clinical conditions to influence the efficacy of auditory gamma entrainment?

S3: Clinical trials

Of the 14 entries retrieved from ClinicalTrials.gov and the WHO ICTRP (Supplementary Data 6), all but one of the trials focus directly on AD or related conditions (i.e. dementia, brain amyloidosis without dementia, individuals with high genetic risk of AD, and individuals with Down syndrome who similarly have an elevated risk of developing AD), with all trials using both auditory and visual gamma stimulation as an intervention. The remaining trial involves a different neurodegenerative condition, Parkinson's disease, additionally employing tactile stimulation. Duration of planned intervention ranges from single session (four trials) to 1-2 months (three trials), to 3-9 months (four trials), to 12 months (three trials). Ten trials employ a randomised, controlled design, three are uncontrolled, and one employs a non-randomised, controlled design. Most of the trials are registered in the USA (12) with just two entries from other countries: Switzerland and Turkey. Ten of the trials include EEG recordings as an outcome measure, while four do not list any measure of entrainment.

Supplementary Data 6. Clinical trials using auditory gamma entrainment for cognitive decline in progress.

The table includes all clinical trial registry entries retrieved that are still in progress. RCT = Randomised controlled trial; AD = Alzheimer's disease; MCI = Mild cognitive impairment; EEG = Electroencephalogram; fMRI = Functional magnetic resonance imaging; MMSE = Mini-mental state examination; ADCS-ADL = Alzheimer's disease cooperative study – activities of daily living; MRI = Magnetic resonance imaging; CDR-SB = Clinical dementia rating – sum of boxes; PET = Positron emission tomography; CSF = Cerebrospinal fluid; RNA = Ribonucleic acid; ADAS-Cog = Alzheimer's disease assessment scale – cognitive subscale; MoCA = Montreal cognitive assessment; UDS V3 = Uniform data set version 3; FAS = Verbal fluency test; GDS = Geriatric depression scale; NPI-Q = Neuropsychiatric inventory questionnaire; PACC = Preclinical Alzheimer cognitive composite; CANTAB = Cambridge neuropsychological test automated battery; CT = Computed tomography.

S4: Quality assessment

Supplementary Data 7. Risk of bias assessment for cross-sectional studies.

The table includes all studies assessed for quality using the AXIS tool (57). Y = Yes; N = No; U = Unknown; N/A = Not Applicable. Low indicates a low risk of bias, moderate indicates a moderate risk of bias, and high indicates a high risk of bias.

Supplementary Data 8. Risk of bias assessment for randomised controlled trials.

The table includes all studies assessed for quality using the RoB 2 tool (7). 1 = Bias arising from the randomisation process; 2 = Bias due to deviations from intended interventions; 3 = Bias due to missing outcome data; 4 = Bias in measurement outcome; 5 = Bias in selection of the reported result. Low indicates a low concern for that domain, some concerns indicate some concern for that domain, and high risk indicates a high concern for that domain.

Supplementary Data 9. Risk of bias assessment for non-randomised trials.

The table includes all studies assessed for quality using the ROBINS-I tool (1). 1 = Bias due to confounding; 2 = Bias in the selection of participants into the study; 3 = Bias in classification of interventions; 4 = Bias due to deviations from intended interventions; 5 = Bias due to missing data; 6 = Bias in the measurement of the outcome; 7 = Bias in the selection of the reported result. Low indicates a low risk of bias, moderate indicates a moderate risk of bias, and high indicates a high risk of bias.

S5. PRISMA 2020 Checklist

Section and Topic	Item #	Checklist item	Reported on Page
**TITLE**			
Title	1	Identify the report as a systematic review.	1
**ABSTRACT**			
Abstract	2	See the PRISMA 2020 for Abstracts checklist.	2
**INTRODUCTION**			
Rationale	3	Describe the rationale for the review in the context of existing knowledge.	3-6
Objectives	4	Provide an explicit statement of the objective(s) or question(s) the review addresses.	6-7
**METHODS**			
Eligibility criteria	5	Specify the inclusion and exclusion criteria for the review and how studies were grouped for the syntheses.	29-30
Information sources	6	Specify all databases, registers, websites, organisations, reference lists and other sources searched or consulted to identify studies. Specify the date when each source was last searched or consulted.	29-30
Search strategy	7	Present the full search strategies for all databases, registers and websites, including any filters and limits used.	29-31
Selection process	8	Specify the methods used to decide whether a study met the inclusion criteria of the review, including how many reviewers screened each record and each report retrieved, whether they worked independently, and if applicable, details of automation tools used in the process.	29-31
Data collection process	9	Specify the methods used to collect data from reports, including how many reviewers collected data from each report, whether they worked independently, any processes for obtaining or confirming data from study investigators, and if applicable, details of automation tools used in the process.	29-31
Data items	10a	List and define all outcomes for which data were sought. Specify whether all results that were compatible with each outcome domain in each study were sought (e.g. for all measures, time points, analyses), and if not, the methods used to decide which results to collect.	29-31
	10b	List and define all other variables for which data were sought (e.g. participant and intervention characteristics, funding sources). Describe any assumptions made about any missing or unclear information.	29-31
Study risk of bias assessment	11	Specify the methods used to assess risk of bias in the included studies, including details of the tool(s) used, how many reviewers assessed each study and whether they worked independently, and if applicable, details of automation tools used in the process.	31
Effect measures	12	Specify for each outcome the effect measure(s) (e.g. risk ratio, mean difference) used in the synthesis or presentation of results.	Tables 1-5
Synthesis methods	13a	Describe the processes used to decide which studies were eligible for each synthesis (e.g. tabulating the study intervention characteristics and comparing against the planned groups for each synthesis (item #5)).	8-10
	13b	Describe any methods required to prepare the data for presentation or synthesis, such as handling of missing summary statistics, or data	9-10

Section and Topic	Item #	Checklist item	Reported on Page
		conversions.	
	13c	Describe any methods used to tabulate or visually display results of individual studies and syntheses.	9-10
	13d	Describe any methods used to synthesize results and provide a rationale for the choice(s). If meta-analysis was performed, describe the model(s), method(s) to identify the presence and extent of statistical heterogeneity, and software package(s) used.	9-10
	13e	Describe any methods used to explore possible causes of heterogeneity among study results (e.g. subgroup analysis, meta-regression).	Table 1
	13f	Describe any sensitivity analyses conducted to assess robustness of the synthesized results.	NA
Reporting bias assessment	14	Describe any methods used to assess risk of bias due to missing results in a synthesis (arising from reporting biases).	31
Certainty assessment	15	Describe any methods used to assess certainty (or confidence) in the body of evidence for an outcome.	31
**RESULTS**			
Study selection	16a	Describe the results of the search and selection process, from the number of records identified in the search to the number of studies included in the review, ideally using a flow diagram.	8
	16b	Cite studies that might appear to meet the inclusion criteria, but which were excluded, and explain why they were excluded.	8
Study characteristics	17	Cite each included study and present its characteristics.	8-17
Risk of bias in studies	18	Present assessments of risk of bias for each included study.	Table 1-5<br>Supplementary Material
Results of individual studies	19	For all outcomes, present, for each study: (a) summary statistics for each group (where appropriate) and (b) an effect estimate and its precision (e.g. confidence/credible interval), ideally using structured tables or plots.	Tables 2-5
Results of syntheses	20a	For each synthesis, briefly summarise the characteristics and risk of bias among contributing studies.	8-10
	20b	Present results of all statistical syntheses conducted. If meta-analysis was done, present for each the summary estimate and its precision (e.g. confidence/credible interval) and measures of statistical heterogeneity. If comparing groups, describe the direction of the effect.	8-17, Tables 2-5
	20c	Present results of all investigations of possible causes of heterogeneity among study results.	8-17
	20d	Present results of all sensitivity analyses conducted to assess the robustness of the synthesized results.	8-17
Reporting biases	21	Present assessments of risk of bias due to missing results (arising from reporting biases) for each synthesis assessed.	Supplementary Material
Certainty of evidence	22	Present assessments of certainty (or confidence) in the body of evidence for each outcome assessed.	Supplementary Material
**DISCUSSION**			
Discussion	23a	Provide a general interpretation of the results in the context of other evidence.	21-32

Section and Topic	Item #	Checklist item	Reported on Page
	23b	Discuss any limitations of the evidence included in the review.	18-27
	23c	Discuss any limitations of the review processes used.	18-27
	23d	Discuss implications of the results for practice, policy, and future research.	18-27
**OTHER INFORMATION**			
Registration and protocol	24a	Provide registration information for the review, including register name and registration number, or state that the review was not registered.	29
	24b	Indicate where the review protocol can be accessed, or state that a protocol was not prepared.	29
	24c	Describe and explain any amendments to information provided at registration or in the protocol.	18-277
Support	25	Describe sources of financial or non-financial support for the review, and the role of the funders or sponsors in the review.	32
Competing interests	26	Declare any competing interests of review authors.	32
Availability of data, code and other materials	27	Report which of the following are publicly available and where they can be found: template data collection forms; data extracted from included studies; data used for all analyses; analytic code; any other materials used in the review.	Supplementary material

References

1. Sahu, P. P. & Tseng, P. Gamma sensory entrainment for cognitive improvement in neurodegenerative diseases: opportunities and challenges ahead. *Front. Integr. Neurosci.* **17**, (2023).
2. Traikapi, A. & Konstantinou, N. Gamma Oscillations in Alzheimer's Disease and Their Potential Therapeutic Role. *Front. Syst. Neurosci.* **15**, (2021).
3. Nimmrich, V., Draguhn, A. & Axmacher, N. Neuronal Network Oscillations in Neurodegenerative Diseases. *NeuroMolecular Med.* **17**, 270–284 (2015).
4. Mably, A. J. & Colgin, L. L. Gamma oscillations in cognitive disorders. *Curr. Opin. Neurobiol.* **52**, 182–187 (2018).
5. Liu, C. *et al.* Modulating Gamma Oscillations Promotes Brain Connectivity to Improve Cognitive Impairment. *Cereb. Cortex* **32**, 2644–2656 (2022).
6. Adaikkan, C. & Tsai, L.-H. Gamma Entrainment: Impact on Neurocircuits, Glia, and Therapeutic Opportunities. *Trends Neurosci.* **43**, 24–41 (2020).
7. Başar, E. A review of gamma oscillations in healthy subjects and in cognitive impairment. *Int. J. Psychophysiol. Off. J. Int. Organ. Psychophysiol.* **90**, 99–117 (2013).
8. Lee, K. *et al.* Optimal flickering light stimulation for entraining gamma waves in the human brain. *Sci. Rep.* **11**, 16206 (2021).
9. Park, Y. *et al.* Optimal flickering light stimulation for entraining gamma rhythms in older adults. *Sci. Rep.* **12**, 15550 (2022).
10. Manippa, V. *et al.* An update on the use of gamma (multi)sensory stimulation for Alzheimer's disease treatment. *Front. Aging Neurosci.* **14**, 1095081 (2022).

11. Parciauskaite, V., Bjekic, J. & Griskova-Bulanova, I. Gamma-Range Auditory Steady-State Responses and Cognitive Performance: A Systematic Review. *Brain Sci.* **11**, 217 (2021).
12. Hajós, M. *et al.* Safety, tolerability, and efficacy estimate of evoked gamma oscillation in mild to moderate Alzheimer's disease. *Front. Neurol.* **15**, 1343588 (2024).
13. Manippa, V. *et al.* An update on the use of gamma (multi)sensory stimulation for Alzheimer's disease treatment. *Front. Aging Neurosci.* **14**, 1095081 (2022).
14. Mockevičius, A., Voicikas, A., Jurkuvėnas, V., Tarailis, P. & Griškova-Bulanova, I. Individualized EEG-Based Neurofeedback Targeting Auditory Steady-State Responses: A Proof-of-Concept Study. *Appl. Psychophysiol. Biofeedback* (2024) doi:10.1007/s10484-024-09662-1.
